# Challenges of nursing students during clinical training: A nursing perspective

**DOI:** 10.3934/publichealth.2024019

**Published:** 2024-03-19

**Authors:** Lobna Mohamed Mohamed Abu Negm, Fathia Ahmed Mersal, Manal S. Fawzy, Ajitha Thankarajan Rajennal, Rehab Salamah Alanazi, Lujain Obaid Alanazi

**Affiliations:** 1 Department of Emergency and Intensive Care Nursing, Faculty of Nursing, Northern Border University, Arar 73213, Saudi Arabia; 2 Department of Public Health Nursing, Faculty of Nursing, Northern Border University, Arar 73213, Saudi Arabia; 3 Unit of Medical Research and Postgraduate Studies, Faculty of Medicine, Northern Border University, Arar 73213, Saudi Arabia; 4 Department of Biochemistry, Faculty of Medicine, Northern Border University, Arar 73213, Saudi Arabia; 5 Department of Medical Surgical Nursing, Faculty of Nursing, Northern Border University, Arar 73213, Saudi Arabia; 6 Faculty of Nursing, Northern Border University, Arar 73213, Saudi Arabia

**Keywords:** challenges, clinical training, nursing education, interpersonal skills development, curriculum alignment, clinical training environment, healthcare professional interaction, NBU

## Abstract

Clinical training plays a fundamental role in nursing students' acquisition of professional capabilities. This study aimed to explore the perceived challenges nursing students face during clinical training. An explorative cross-sectional study was applied. A proportionate, stratified, random sample was enrolled in the study with inclusive criteria, including nursing students (2nd - 4th year) and interns who attended their internship in regional hospitals. A validated electronic questionnaire was used for data collection, which consisted of three sections and 29 items. The section that focused on the difficulties experienced by nursing students during their practical training included six elements: teachers, healthcare professionals, the students themselves, tasks, time management, and the location of the training. Another section inquired about the students' perspectives on the benefits of clinical training. A three-point “Likert scale” was applied. The findings indicated that mild (24%), moderate (62%), and severe (14%) degree of challenges were perceived by the study participants. The mean score for the total challenges during clinical training was 2.00 ± 0.28, and there were variations in the perceived challenges among grade levels. In conclusion, there are variations in the perceived challenges during clinical training among different grade levels. These challenges were related to teachers, health workers, the students, tasks, the time, and the place. Enhancing the nursing curricula alignment with practical training objectives is recommended, focusing on the development of technical and interpersonal skills with appropriate guidance, alongside positive clinical settings to help nursing students learn and boost their confidence in their approach.

## 1. Introduction

Educational experiences in a clinical setting, where both faculty members and students actively participate, are critical to the training of nursing students [1]. Nevertheless, numerous elements, including the clinical context and the syllabus, can influence a student's progression. Understanding these elements and their intricate associations provides a comprehensive approach to students' needs. It is essential to obtain insights into these elements to effectively address possible difficulties [2].

Clinical education settings, which is a phrase commonly applied in nursing instruction, refers to any circumstances where nursing learners apply their theoretical knowledge to practical use through actual or simulated patient care. Such educational settings present invaluable occasions for nursing students to garner the comprehension, abilities, and practice essential to render high-quality care [1].

Clinical training targets nurture and evolve students' professional skills to adequately provide clinical healthcare [2]. However, they confront diverse hurdles such as the scarcity of resources, skilled personnel, and the lack of educational access. A substantial portion of the nursing course—approximately half of the educational timeline—is allocated to clinical training. These obstacles can complicate a nurse's ability to deliver suitable care and can potentially create a hazardous educational ambiance [3].

A clinical faculty member's role in the development and growth of nursing students is pivotal. These faculty members assist students to hone their clinical judgment and draw associations between theoretical knowledge and hands-on practice. Consequently, they are perceived as the fundamental link between learners, healthcare professionals, and patients. To optimally perform their responsibilities, these faculty members must possess certain essential professional traits such as comprehensive professional knowledge, proficiency in clinical procedures, excellent communication skills, and the ability to be exemplary role models [4]. Faculty members must communicate well, anticipate and respond to student feedback and needs, and demonstrate proficiency in both in-person and online pedagogical methodologies [5].

There are a myriad of elements that contribute to the successful incorporation of theoretical knowledge into practical use. Among the key factors are an individual's readiness on both physical and emotional levels, their educational background, as well as their hands-on clinical exposure [6]. Additionally, the availability of supportive resources, instructional materials, and the implementation of potent teaching methods play essential roles. Further factors can involve the quality of the learning setting, the rapport between the student and the teacher, and the student's motivation level [7]. In addition, various personal aspects can impact merging theoretical concepts with hands-on work. Such elements encompass the student's depth of knowledge, their competency in implementing theory in practical scenarios, and their capacity to combine their knowledge with their skill sets. Importantly, each factor has the potential to either enhance or impede the process by which nursing students gain their clinical abilities [8]. On entry into the clinical environment, nursing students are confronted with many challenges and problems that affect their learning [9],[10].

Numerous factors can influence clinical learning, including the nature of supervision and feedback and the traits of the faculty members and students. Moreover, there is a noticeable shortfall in comprehensive research concerning clinical teaching - an integral part of professional nursing education for students [11]. The effectiveness of Saudi Arabia SA's clinical teaching leaves much to be desired, signaling an immediate need for more engaging clinical settings. These settings would breathe life into theoretical concepts as applicable practices, thus motivating students. Several hurdles affect teaching in the clinical environments of Saudi Arabia [12]. A qualitative descriptive study about nursing education challenges from Saudi nurse faculty members' and leaders' perspectives revealed that participants stated that nursing education in Saudi Arabia encounters numerous obstacles that demand significant focus from Saudi policymakers. The problems were categorized into four themes: cultural, educational, organizational (including inadequate nursing authority and lack of acknowledgment for Saudi nurses), and job challenges (such as poor working conditions and language barriers) [13]. Time constraints, an issue faced universally, are one critical challenge. Additionally, there may be several potential issues and deficiencies that could obstruct the development of an optimal environment for clinical teaching [14].

The effective collaboration between academic institutions and healthcare facilities is crucial to address the issues faculty members encounter when working in clinical teaching environments with nursing and medical teams. Communication transparency between these entities is vital for the productivity and success of partnerships. This could be facilitated through routine rendezvous and constructive critiques. Aptly preparing students to interact with healthcare professionals and teams is equally indispensable. Delivering the appropriate resources, such as books and lectures, alongside practical exposure within clinical surroundings, are valuable tools for preparation. Moreover, it is essential to have a mechanism that promotes information exchange, such as faculty members frequently updating healthcare facilities with reports. Through profound communication and partnerships between educational institutions and hospitals, we can strive to furnish students with exceptional clinical education [15]. Clinical placements can enhance the quality of nursing and higher education within healthcare by improving students' academic performance and providing practical work experience. Close coordination between higher education institutions and healthcare organizations is crucial to ensure the quality and success of clinical placements [16].

Healthcare professional training programs usually mandate clinical, workplace-based trainings before obtaining a bachelor's degree. Clinical education primarily occurs in teaching hospitals, making academic collaborations between universities and teaching hospitals essential to progress and maintain clinical education [17].

Collaboration between healthcare institutions offers opportunities for institutional updates and renewal through information and professional knowledge exchange. Additionally, it helps graduate students earn professional recognition by providing a conducive working environment. However, effective planning is crucial for successful collaborations. This dialogue allows for the selection of suitable clinics, provision of materials, effective communication, and safety for students and patients. However, current plans show that communication between the two institutions is not at the desired quality, and protocols are only structured to show student dates and locations [18].

Nursing education is crucial for nursing professionals, and clinical training is a key component [1]. However, students often face challenges during clinical training, thus affecting their learning experience [19]. Identifying and addressing these challenges is essential for effective clinical training programs and is crucial to design interventions and implement improvements at the Nursing College, Northern Border University, thus enhancing the educational experience, and equipping students with the necessary skills. The literature suggests a lack of research exploring the difficulties nursing students face during their clinical training in our region [20]. Therefore, this study aims to investigate the difficulties experienced by nursing students in their clinical training to enhance the effectiveness of clinical education at the Nursing College of the Northern Border University.

## 2. Materials and methods

### 2.1. Research design

An explorative cross-sectional study was used to conduct this study. The challenges of nursing students were evaluated using a validated questionnaire to assess challenges about teacher-related factors, patient-related factors, performance tasks-related factors, and time/place-related factors.

### 2.2. Ethics approval of research

An ethical agreement was obtained from the Local Bioethical Committee (HAP-09-A-043) at Northern Border University (approval No. 76/44/H) dated 03/09/2-23. Furthermore, the administrative agreement was obtained from the dean of the colleges included in the study. Consent was achieved from the participant before their inclusion in the study and after explaining the aim of the study. The participants were informed that all information was coded and confidentiality was kept.

The study involved students who provided their consent, which is a standard practice in research involving human participants. To prevent coercion, researchers explained the study's purpose, objectives, and methodology, including potential risks and benefits. Students could also ask questions or express concerns before consent, allowing them to make informed decisions. Participation in research studies is typically voluntary, and students can withdraw their consent at any time without negative consequences. Researchers should have emphasized this to empower students to make their own decisions. Ethical guidelines and institutional review boards (IRBs) oversaw research involving human participants to ensure ethical standards were upheld and participants were adequately protected.

### 2.3. The target study population

Nursing students at the local University present at the time of the study were approached to be included, with a proportionate stratified random sampling technique. The inclusion criteria included female and male nursing students (2nd - 4th year) and interns who attended their internship in the local region and were willing to participate in the present study. The sample size of 298 nursing students at Northern Border University contained 168 eligible participants to be recruited to achieve a confidence level of 95%, with type I error (a) = 0.05, type II error (B) = 0.2, and a power of 0.80.

$$n=\frac{N\times p(1-p)}{\left[N-1\times \left(d^{2}÷z^{2}\right)\right]+p(1-p)}$$ (1)

The proportionate stratified random sampling was achieved as follows: 67 students within their second year, 47 students within their third year, 29 students within their fourth year, and 25 students within the internship, where the total number of participants was 168.

### 2.4. Exclusion criteria

To ensure that the study's results were not influenced by factors such as a lack of experience and geographical differences and to align with the ethical regulations, first-year nursing students (without clinical training), interns who completed their internships outside of our region, and unwilling students were excluded from the study.

### 2.5. Data collection

The researchers distributed the structured questionnaire on Google Forms to gather the student's perspectives about their challenges during clinical training. The questionnaire was rigorously developed, entailing both adaptation from existing validated scales and de novo item generation [21]–[24]. The adapted questionnaire underwent a comprehensive translation and cultural adaptation process, thus ensuring contextual relevance and comprehension for our target Arabic-speaking participants. The process was meticulously documented to maintain the integrity of the questionnaire's construct. A three-point Likert scale was employed, with response options ranging from “always,” “sometimes,” to “never.” This survey was comprised of five sections and 29 questions. The first section focused on the student's demographic information, including age, gender, and academic year. The second and third sections explored teacher-related (six statements) and patient-related (five statements) factors and their impact on nursing students' training experience. The fourth section delved into the challenges faced by nursing students concerning their performance tasks (eight statements). Lastly, the fifth part explored time and place-related factors, thereby investigating the challenges that arise in terms of timing and location during nursing students' practical training. This section encompassed ten statements.

### 2.6. Scoring system

The total challenges of nursing students during clinical training were scored as follows according to a weighted mean: mild challenge (= 1.00–1.66), moderate challenge (= 1.67–2.32), and severe challenge (= 2.33–3.00).

The weighted mean scoring system is a standardized method used in nursing education and research to assess challenges faced by nursing students during clinical training. This method is widely accepted and provides a reliable and consistent method to quantify the severity of these challenges. It has been widely used in various research studies [25],[26].

### 2.7. Validity and reliability

The researchers adapted, translated, and modified a data collection tool that was tested for content and face validity by five professionals in the field of nursing. The reliability test was performed using the “Alpha Cronbach's” test, whereas the challenges of nursing students during the clinical training tool consisted of 29 items with 0.840 Alpha Cronbach's, which revealed a good reliability.

### 2.8. Preliminary study

A pilot study was conducted on 10% of the participants to test the clarity, validity, reliability, and feasibility of the research process, the applicability of the tools, the maneuvers of the interventions, and to estimate the time needed to answer the questionnaire. The final forms were created and modified, and various details were omitted based on the results. Participants in the pilot study were not involved in the study sample.

### 2.9. Statistical analysis

The collected data was revised, tabulated, and analyzed using IBM SPSS Statistics for Windows, Version 26 (Released 2019; IBM Corp., Armonk, New York). Shapiro-Wilk and Kolmogorov-Smirnov tests were used to determine the data distribution. When appropriate, descriptive and comparative statistics as numbers (frequencies) and percentages, mean and standard deviations (*Mean* ± *SD*), Chi-square (*χ^2^*), Kruskal Wallis, and Mann-Whitney tests were applied. Statistical significance was considered at *p*-value < 0.05.

## 3. Results

### 3.1. Demographic characteristics of the study population

The demographic characteristics of the study participants are depicted in Table 1. It shows that about 62% of the nursing students were under the age of 20. The participants had an average age of 19, with a standard deviation of 1.12, and one-third (30%) were male. Regarding their academic level, 16% were in their second year, 26% were in their third year, and the majority (43%) were in their fourth year. Moreover, the internship year was undertaken by 15% of the participants.

**Table 1.** Demographic characteristics of the study participants.

**Items**	**No.**	**%**
**Age/years**		
<20	131	62.1
≥20	80	37.9
***Mean* and *SD***	19.42 ± 1.12
**Sex**		
Male	64	30.3
Female	147	69.7
**Grade level**		
Second	34	16.1
Third	55	26.1
Fourth	90	42.6
Internship	32	15.2

Note: Data (except age) are presented as numbers (No.) and percentages (%). The total number of study participants (*n* = 211).

### 3.2. Challenges during clinical training faced by the nursing student

Table 2 comprehensively assesses various factors influencing student learning and practical training in a clinical setting. It identifies and quantifies four major input categories: teacher-related factors, patient-related factors, performance-related factors, and time/place-related factors.

In the category of teacher-related factors, the majority of nursing students reported concerns about the teacher-student ratio, with 34% stating that the ratio was not suitable for the number of students. Nearly two-thirds (66%) of students reported, “Do not allow entrance to some units and wards.” Regarding the performance-related factors, students reported that 38% always felt fear, embarrassment, and indecision. Meanwhile, concerning time/place-related factors, such as insufficient rest periods, 54% reported it as “always”. These findings highlight the need for adjustments to improve nursing training.

When applying a scoring system on the previously mentioned four domains of the challenges encountered during clinical training (Table 3), nearly 53% of nursing students identified the teacher-related factors as mildly challenging, and 47% characterized them as moderately challenging. The mean score for these teacher-related factors was calculated at 1.97 ± 0.22.

Regarding patient-related factors, about 32% of nursing students experienced mild challenges, and 60% experienced moderate challenges. The mean score for patient-related factors was 1.83 ± 0.41. Performance task-related factors were considered a mild challenge by 22% of the students and a moderate challenge by 65.9%. The mean score for these factors stood at 1.96 ± 0.32.

Time and place-related factors encompassed a severe challenge for 32% of the participants and moderate for 49% of nursing students. Here, the mean score achieved was 2.15 ± 0.53.

**Table 2.** Nursing student response regarding challenges faced during clinical training.

**Items**	Always		Sometimes		Never		** *x* **	***SD***
	**No**	**%**	**No**	**%**	**No**	**%**		
**Teacher related factors**								
Teachers' number is unsuitable with students' number	20	9.5	119	56.4	72	34.1	1.75	0.62
The teacher gives attention (care) to the practical training time	106	50.2	99	46.9	6	2.8	2.48	0.56
A teacher does not follow up with students during practical training directly	11	5.2	100	47.4	100	47.4	1.58	0.59
The teacher follows up with students depending on restorative (supervisor)	49	23.2	128	60.7	34	16.1	2.07	0.63
The teacher connects theoretical subjects with practical application	78	37.0	116	55.0	17	8.1	2.29	0.61
The theoretic subject does not synchronize with the practical subject	8	3.8	120	56.9	83	39.3	1.64	0.55
**Patient-related factors**								
The collaboration of nursing staff with students was not sufficient	42	19.9	107	50.7	62	29.4	1.90	0.69
The collaboration of medical staff with students was not sufficient	42	19.9	108	51.2	61	28.9	1.91	0.69
Language is a barrier to communicating with patients	10	4.7	116	55.0	85	40.3	1.64	0.57
Don't allow entrance to some units and wards	139	65.9	18	8.5	54	25.6	1.83	0.56
The presence of more than one relative with the patient causes embarrassment to the student during practice	27	12.8	124	58.8	60	28.4	1.84	0.63
**Performance-related factors**								
The absence of a student affects his/her application to nursing practice	68	32.2	123	58.3	20	9.5	2.23	0.61
Student dislikes nursing practices application	5	2.4	90	42.7	116	55.0	1.47	0.55
Gender differences (for students) affect communication with patients	21	10.0	118	55.9	72	34.1	1.76	0.62
Tools and equipment not available	37	17.5	128	60.7	46	21.8	1.96	0.63
Medical machines (monitors, DC shock, ECG, etc.) do not work effectively.	30	14.2	106	50.2	75	35.5	1.79	0.68
The presence of a high number of students (institution, schools) decreases the opportunity for application and practice	52	24.6	106	50.2	53	25.1	2.00	0.71
Fear, embarrassment, and indecision decrease practical opportunities	80	37.9	115	54.5	16	7.6	2.31	0.61
The presence of personal problems among students decreases their performance	64	30.3	112	53.1	35	16.6	2.14	0.68
**Time and place-related factors**								
PA practical day's number in a week is tiring	58	27.5	72	34.1	81	38.4	1.89	0.81
Practical hour's numbers are tired	83	39.3	78	37.0	50	23.7	2.16	0.78
Standing for long hours inward for data collection from the patient	98	46.4	84	39.8	29	13.7	2.33	0.71
The rest period is insufficient	115	54.5	69	32.7	27	12.8	2.42	0.71
Far of training places and numerous of it	83	39.3	89	42.2	39	18.5	2.21	0.74
Hospital capacity is not enough for training	70	33.2	75	35.5	66	31.3	2.02	0.81
Wards design in the hospital is inappropriate with clinical training	70	33.2	77	36.5	64	30.3	2.03	0.81
Impossibility of receiving study halls for lecture viewing scientific films	78	37.0	88	41.7	45	21.3	2.16	0.75
Unavailability of a specific place to keep personal things	122	57.8	56	26.5	33	15.6	2.43	0.75
Ambulate media is not obtainable	61	28.9	56	26.5	94	44.5	1.85	0.85

Note: Data are presented as numbers, percent, mean (x), and standard deviation (SD). The total number of study participants (*n* = 211).

To summarize, the challenges during the clinical training were classified as mild by 24% of nursing students and moderate by the majority (62%). Hence, the mean score for total challenges during clinical training was 2.0 ± 0.28.

**Table 3.** Scoring of nursing student response regarding challenges faced during clinical training.

**Items**	**Mild challenge**		**Moderate challenge**		**Severe challenge**	
	**No**	**%**	**No**	**%**	**No**	**%**
**Teacher related factors**	111	52.6	99	46.9	1	0.5
*Mean* and *SD* of Teacher related factors	1.97±0.22
**Patient-related factors**	67	31.8	127	60.2	17	8.1
*Mean* and *SD* of Patient-related factors	1.83±0.41
**Performance-related factors**	47	22.3	139	65.9	25	11.8
*Mean* and *SD* of Performance-related factors	1.96±0.32
**Time and place-related factors**	40	19.0	103	48.8	68	32.2
*Mean* and *SD* of Time and place related factors	2.15±0.53
**Total Challenges During Clinical Training**	51	24.2	130	61.6	30	14.2
*Mean* and *SD* of Total Challenges during Clinical Training	2.0±0.28

### 3.3. Relation of demographic characteristics and challenges faced by the nursing students

The correlation between demographic facets and the obstacles nursing students faced during their clinical training is depicted in Table 4. Evaluating age as a factor, we found that nursing students under 20 reported a mean challenge score of 1.89 ± 0.29. Contrarily, nursing students aged 20 and above exhibited a considerably higher mean challenge score of 2.03 ± 0.33. This shows a significant discrepancy between the two age categories with a *p*-value of 0.001. Meanwhile, there was no observed significant difference regarding the gender and the mean score of the challenges nursing students faced during their clinical training.

Concerning the academic year, second-year nursing students noted mean challenge scores of 2.01 ± 0.35 during clinical training. In contrast, third-year nursing students reported a notably lower mean score, standing at 1.79 ± 0.25. Meanwhile, fourth-year nursing students recorded marginally increased mean challenge scores at 1.98 ± 0.32. This implies a significant variation in the perceived challenges during clinical training across different academic years, as validated by a *p*-value < 0.001. These findings indicate distinctive experiences and challenges based on the demographic attributes of nursing students during their clinical training.

**Table 4.** Relation of demographic characteristics and challenges of nursing students during clinical training.

**Parameter**	**Total challenge**		***Z/H*-test**	***p*-value**
	** *x* **	***SD***		
**Age/ years^a^**				
<20	1.89	0.29	Z = -3.35	0.001*
≥20	2.03	0.33		
**Sex^a^**				
Male	1.95	0.33	Z = -0.37	0.715
Female	1.94	0.31		
**Grade^b^**				
Second	2.01	0.35	H = 21.44	<0.001*
Third	1.79	0.25		
Fourth	1.98	0.32		
Internship	2.04	0.26		

Note: ^a^ The *p*-value has been calculated using the Mann-Whitney Z-test. ^b^ The *p*-value has been calculated using the Kruskal Wallis H-test. * Significance was set at *p* < 0.05. x: mean, *SD*: standard deviation.

## 4. Discussion

Early clinical experiences are crucial for students' training, providing unique opportunities for personal and professional growth. These experiences promote relationships, education with patients, and integration with the curriculum. Additionally, they promote motivation, confidence, and satisfaction, thus contributing to the development of their professional identity. These experiences help students apply their knowledge, develop interpersonal skills, and appreciate patient-centered care [27]. In Saudi Arabia, nursing students undergo clinical training using a structured model that combines classroom instruction and hands-on clinical experience [28]. The crucial role of the clinical learning setting in determining the caliber of nursing education has been globally acknowledged. Despite this, existing literature highlights significant issues concerning both theoretical and practical training aspects. Such issues have tangible impacts on student learning, often imposing difficulties within their clinical training environments [29]–[32]. Despite the global recognition of education, there are significant issues in both theoretical and practical training aspects, which can impact student learning and create difficulties within clinical training environments [33]. These issues include inadequate resources, limited access to clinical placements, a high student-to-instructor ratio, outdated curriculum, lack of interprofessional collaboration, and inadequate assessment methods [34]. Insufficient resources can hinder students' ability to acquire the necessary knowledge and skills in their field, while limited access to clinical placements can restrict exposure to real-world clinical scenarios [35]. A high student-to-instructor ratio can result in limited individual attention and feedback, thus challenging personalized guidance [36]. Outdated curriculum content can lead to a mismatch between the knowledge and skills taught and the current requirements of the profession, potentially limiting students' preparedness for real-world clinical scenarios [37]. A lack of interprofessional collaboration can hinder students' ability to develop effective communication and collaboration skills, which are crucial for providing comprehensive and coordinated patient care [38].

The findings of this study revealed that around two-thirds of nursing students were younger than 20 years old, while more than one-third were 20 years old or older. The mean age of the participants was 19.42 ± 1.128. Concerning gender, less than one-third of the students were male, while the remaining students were female. Regarding the grade level, less than one-fifth of participants were in their second year, more than a quarter were in the third grade, and more than two-fifths were in the fourth grade. Additionally, more than one-tenth of the participants were in the internship year. A study by Alanazi et al. in a similar environmental context in Saudi Arabia reported comparable participants' characteristics [39].

The present study assessed various factors that influenced student learning and practical training in a clinical setting. It identified and quantified four significant categories: teacher-related factors, patient-related factors, performance-related factors, and time/place-related factors. In the category of teacher-related factors, the majority of nursing students reported concerns about the teacher-student ratio, stating that the ratio was not suitable for the number of students. Ensuring an optimal student-teacher ratio is essential in clinical environments to provide proper monitoring, personalized guidance, and patient safety. In our college, a ratio of 17 students to 1 educator indicates a greater group size, potentially affecting the amount of individual attention and supervision each student gets. Nearly two-thirds of students reported, “Do not allow entrance to some units and wards.” Concerning performance-related factors, more than one-third of students always felt embarrassment and indecision. Regarding time and place-related factors, such as insufficient rest periods, more than one-half of students reported it as “always.” These findings highlight the need to improve nursing education consistent with others [31],[40],[41]. Drateru reported a comprehensive overview of the multifaceted challenges encountered by student nurses during clinical skills acquisition [31]. This report identified several constraints within the clinical environment, such as limited access to certain wards and units, psychological barriers including embarrassment and indecision that students often face, and systemic issues such as inadequate rest periods. These obstacles to learning crucially impact the educational experience and align with the concerns reported by our participants. Moreover, Günay and Kılınç highlighted the apprehension of students to engage hands-on with patients and their self-perceived deficiencies in translating theoretical learning into actual clinical skills. These concerns, coupled with reported issues such as challenging hospital environments, insufficient mentorship, and disproportionate expectations, echo the struggles we identified among students who frequently encountered humiliation, indecisiveness, and a lack of adequate rest [40]. Furthermore, the Carless-Kane and Nowell review reinforced the obstacles delineated by our work, such as the disconnection between academic coursework and real-world clinical applications, as well as the critical need for supportive and knowledgeable faculty members to facilitate an effective learning transfer [41]. Their review underscored a multitude of factors that either aided or impeded this essential transition from theoretical understanding to practical application—factors that are prominently echoed in our study's reported experiences of nursing students who encountered difficulties in applying learned concepts within clinical settings and required improved institutional support and educational strategies to bridge this transitive gap. A flourishing clinical learning ambiance can only be actualized through the concerted efforts of faculty members and administrative personnel to provide students with unhindered access to essential resources and requisite support [42]. Moreover, it is pivotal for nursing faculty members to consider the unique requisites of their student cohort to curate a clinical learning environment that will supply an optimal educational experience [1].

Clinical faculty members possess advanced degrees in nursing, clinical expertise, and professional development programs. They have practical experience in their specialization and undergo training to promote student engagement, critical thinking, and clinical competence. Continuous professional development is essential for clinical faculty members to stay updated with the latest advancements in nursing education, clinical practice, and healthcare. They may attend conferences, participate in research projects, or pursue additional certifications or credentials. Regarding teacher-related factors of challenges faced by participants during clinical training, our study revealed that the lowest score was “A teacher does not follow up with students during practical training directly,” followed by “Theoretic Subject does not synchronize with the practical subject.” This finding agreed with the recent findings of Attia and Ibrahim [15], who found that most students reported having a successful type of supervision. Similarly, Abuosi et al. [43] concluded that successful supervision is essential for students to get the most out of their clinical learning environment. This is congruent with Stevens's conclusion that integrating theory into practice is an essential component of clinical skills acquisition among nursing students [44]. The ability to effectively translate knowledge into practice is a critical factor to achieve successful outcomes and patient safety.

Clinical nurses are essential to provide supportive relationships to students in their transition to a healthcare setting. This support can help foster an environment of acceptance, thus allowing students to quickly learn the necessary skills to become a successful part of the treatment team [45]. On the other hand, Hanifi et al. [46] stated that staff and student relationships are often a source of tension in clinical learning environments. This can lead to poor communication and a lack of trust, respect, and understanding between the two groups. These tensions can be detrimental to the clinical learning process, as they can lead to feelings of dissatisfaction and mistrust between the staff and students, which can then lead to a breakdown in the learning process. To ensure the best learning experience for the staff and students, the relationship between the two groups must be managed to promote trust and respect. This can be done by establishing clear communication channels and implementing appropriate policies and procedures that promote a safe and productive learning environment [47].

The present study found that the highest mean score related to patient factors was “Collaboration of nursing staff with the student was insufficient,” while the lowest score was “Language is a barrier in communicating with the patient.” This finding was consistent with Alamri and Sami [48], who revealed that the study participants communicated dissatisfaction regarding the insufficient support from registered nurses during their hospital internships. Specifically, the nursing students reported unsupportive instances of specific faculty nursing staff at the hospital during their skill practice sessions. The relationship between educational institutions and healthcare systems is crucial, aimed at tackling the difficulties experienced by nursing students during their clinical education, especially while receiving hands-on training from nurses and medical personnel. A recent study by Banafi reported that nursing students of the researcher's University presented good knowledge and positive attitudes toward English medical writing skills [49].

Regarding performance tasks-related factors, it showed that the highest mean was “Absences of student effect on his/her application to nursing practice,” followed by “Presence of personal problems among students decrease students' performance.” Clinical education programs require students to complete three credit hours for each clinical area, resulting in six actual hours. These hours may vary depending on the program and regulatory guidelines. If students miss clinical hours due to illness or personal circumstances, educational programs may have policies for makeup sessions, scheduling additional hours, or finding alternative opportunities. These policies and procedures depend on the institution and program. Avoiding complex patient groups can limit exposure to diverse patient populations and hinder learning and professional growth. In some cases, students may be required to complete additional clinical hours or specific assignments to meet learning outcomes. Alternatively, they may receive guidance from clinical faculty members to address any concerns or challenges in caring for specific patient groups. The Magobolo and Dube study suggested that nursing students at the chosen institution typically missed their clinical assignments due to personal illness and familial issues, as well as financial constraints that arose from their education being funded but their clinical work not being compensated [50]. Students aged 18 to 30 were more likely to avoid specific wards populated with ill patients, further contributing to absenteeism. Furthermore, concrete evidence highlighted that student absenteeism in the clinical workplace could also be attributed to an excessive workload, stepping in to fill staffing gaps, or experiencing maltreatment from senior staff members. As a result, student nurse absenteeism was prominent in the clinical environment [38]. A recent report by Akkus and Cinkir confirmed that student absenteeism remains one of the significant obstacles for educational organizations to achieve their goals and adversely affects the academic and social development of the student [51].

Currently, the highest mean related to time and place factors was “The unavailability of a specific place to keep personal things,” while the lowest score was “Ambulate media is not obtainable.” This aligns with a previous study by Yaseen Fathi and Ibrahim [3], who stated that limited access to resources and equipment hindered students' learning and ability to apply the concepts they were learned in the classroom. To address this issue, the school implemented several initiatives and strategies to provide the students with the necessary resources and equipment to gain the knowledge and experience needed.

Our study highlights the varying difficulties nursing students face during their clinical training due to faculty member-related factors. Over half of the students reported experiencing mild challenges, closely followed by less than half of the students who expressed moderate challenges, and a very low percentage underwent severe challenges. The average score for teacher-related challenges was 1.97 ± 0.22. In a recent study conducted in a similar learning environment exploring “the factors affecting nursing students clinical learning from student viewpoint,” most students found their faculty members' personalities complex. Additionally, 92 students felt a disparity in the student-to-faculty member ratio. About 86 students claimed their faculty members seemed more focused on their appearance compared to their educational competence. Eighty-five students critiqued their faculty members for not utilizing updated training methods, and there were also complaints regarding the faculty members' communication skills, as stated by 79 participants [52].

Concerning patient-related factors, the current study demonstrated a mild challenge reported by less than one-third of nursing students, a moderate challenge reported by more than half of nursing students, and a severe challenge reported by less than one-tenth. The mean score for patient-related factors was 1.83 ± 0.41. This finding was congruent with Alotaibi et al. [53], who stated that challenges faced by nursing internship students, such as technical and procedural difficulties, communication and decision-making issues, and personal well-being and safety concerns, highlight the need for nursing education programs to focus more on preparing students for the complexities of emergency care. Additionally, Alamri and Sami unraveled the problems and challenges encountered by nursing students during their internship program at a hospital in Medina, Saudi Arabia, and demonstrated that nursing interns experienced issues related to clinical practice. These issues included a lack of support from nurses and health professionals, inadequate training, an apparent distrust of students by nurses and physicians about patient care, and the absence of preceptorship to guide during the internship program [48].

Concerning performance task-related factors, our research showed a mild challenge reported by less than a quarter of nursing students, a moderate challenge reported by nearly two-thirds of nursing students, and a severe challenge reported by a tenth of them. The mean score for performance task-related factors was 1.96 ± 0.32. A study published in 2022 regarding factors affecting nursing students' learning in clinical venues from a student viewpoint at Taif University reported that most nursing students agreed that they worried about making mistakes in clinical placements. They noted a lack of field follow-up incorporated into the training regimen. Additionally, the absence of defined daily objectives was highlighted. Additionally, concerns were raised about an inability to effectively interact with patients due to the training. Furthermore, they voiced their disappointment over not being informed about the goals of the hands-on training and indicated a lack of preparedness for such practical training sessions [52].

For time and place-related factors, our study revealed mild (19%), moderate (49%), and severe (32%) challenges reported by the nursing students. The mean score for this item was 2.15 ± 0.53. Our findings align with those shared by Ugwu, Ogbonnaya, Chijioke, and Esievo in 2023. Their findings illustrated that a small group within the study highlighted a short semester and limited clinical placement duration as factors resulting in an insufficient length of clinical postings and a lack of exposure to specialty fields. This reportedly affected the degree of proficiency in specific procedures from these specialties due to a lack of practical experience, further exacerbating the gap in those under-practiced clinical procedures. Similarly, a qualitative study centered around the obstacles nursing students face in their clinical learning environment outlined that nursing students tend to have limited opportunities to sufficiently repeat and improve these skills before fully integrating into the clinical setting [29].

Overall, the total challenges during clinical training were mild in one-quarter of nursing students, moderate in less than two-thirds of them, and severe in more than one-tenth of nursing students, with a mean score of 2.0 ± 0.28. Anarado et al. showed that many factors could hinder the clinical training of students in nursing educational institutions in Southeastern Nigeria [54]. These factors included a lack of qualified personnel to teach and mentor students, inadequate or outdated equipment, a lack of clinical opportunities, and inadequate funding. The study also highlighted a need for improved communication between faculty and students and improved student access to clinical resources [54]. These findings confirm the current results, which suggest that several factors can impede clinical training and lead to poorer student outcomes.

Upon examination of the association between demographic characteristics and the issues faced by nursing students during clinical training, it was observed that younger students (<20 years) reported a mean challenge score of 1.89 ± 0.29. Conversely, nursing students ≥20 years demonstrated a higher mean total challenge score of 2.03 ± 0.33, thus indicating a considerable difference between the two age groups. Similarly, Ergol and AkyÜZ deduced that most participants grappled with issues during their clinical practice associated with the nurses, hospital environments, and theoretical education. Half of these participants believed that their difficulties could be partially surmounted. A majority could moderately apply their classroom learning to their clinical practice. All participants who reported a particular incident that impacted them in a clinical setting cited an unfavorable event, most linking these events to nursing attitudes and behaviors [55].

Regarding the academic year, there were differences in the perceived hurdles during clinical training across different grades among nursing students. This observation is inconsistent with Aljohani et al. [56], who discovered that nursing students in their third year of their bachelor's degree experienced the most stress related to clinical challenges, in contrast to first-year students who experienced the least. The high scores in intellectual challenge among third-year nursing students might be tied to the compulsory coursework in their clinical training, such as adult medical-surgical nursing, pediatric nursing, and courses in maternity nursing [44].

The present study highlights the importance of understanding the teacher-student ratio, access to units and wards, performance-related factors, time and place-related factors, and demographic differences in challenges faced by nursing students. Many students expressed concerns about the ratio, suggesting further research on its impact on student satisfaction, learning outcomes, and patient safety. Moreover, limited access to specific units and wards is a concern, which require strategies to address this issue. Performance-related factors, such as fear, embarrassment, and indecision, should be studied to improve a student's performance and confidence. Time and place-related factors, such as insufficient rest periods, should be addressed to optimize rest periods and enhance the learning environment. Demographic differences in challenges, such as age and academic year, should be further explored to inform targeted interventions and support mechanisms.

## 5. Study limitations

The possible limitations of the current study are as follows:

(1) Sample selection bias: the study primarily includes nursing students from regional hospitals only. Results might not generalize to nursing students in other community hospitals.

(2) Response Bias: Since the study relies on self-reported data gathered through questionnaires, participants might have responded in a way they perceived as socially desirable, rather than providing accurate responses that reflect their experiences.

(3) Limited scope of the “three-point Likert Scale” that could potentially limit the respondents' capability to express their feelings or opinions accurately.

(4) Using a cross-sectional design could limit the ability to establish a clear cause-and-effect relationship between identified challenges. Longitudinal studies can aid in mapping progress and changes over time, thus providing more insightful results.

(5) Lack of consideration of other potential variables such as student mental health, resource availability, and cultural factors that might impact the perceived challenges.

(6) Potential variations in internship settings at different hospitals and clinics might have different environments, resource availability, mentorship quality, etc. If these variations are not considered, they might affect the interpretation of the results.

## 6. Conclusions

The present research findings indicated that facets related to patients and performance tasks posed a moderate to high challenge for nursing students during their clinical training in this region. Factors related to the faculty members posed only minor difficulties. Additionally, age and the academic year played a role in the difficulties reported, with older students and those in higher grades reporting more challenges. Among all, place-related elements were the most challenging, suggesting that the suitability of the clinical site and availability of time might act as hurdles in the learning process of nursing students.

## Use of AI tools declaration

The authors declare they have not used Artificial Intelligence (AI) tools in the creation of this article.

## References

[1] Zhang J, Shields L, Ma B (2022). The clinical learning environment, supervision and future intention to work as a nurse in nursing students: A cross-sectional and descriptive study. BMC Med Educ.

[2] Farzi S, Shahriari M, Farzi S (2018). Exploring the challenges of clinical education in nursing and strategies to improve it: A qualitative study. J Educ Health Promot.

[3] Yaseen Fathi K, Ibrahim RH (2023). Factors influencing integration of theory into practice in clinical skills acquisition among nursing students. Inform Med Unlocked.

[4] Zhang J, Zhou F, Jiang J (2022). Effective Teaching Behaviors of Clinical Nursing Teachers: A Qualitative Meta-Synthesis. Front Public Health.

[5] Liesveld J, Rohr J, Petrovic K (2023). Nursing student challenges during the COVID-19 pandemic from 2020 to 2021: A thematic analysis. Teach Learn Nurs.

[6] Lee T, Lee SJ, Yoon YS (2023). Personal Factors and Clinical Learning Environment as Predictors of Nursing Students' Readiness for Practice: A Structural Equation Modeling Analysis. Asian Nurs Res (Korean Soc Nurs Sci).

[7] Lawrence JE, Tar UA (2018). Factors that influence teachers' adoption and integration of ICT in teaching/learning process. Educ Media Int.

[8] Wrenn J, Wrenn B (2009). Enhancing learning by integrating theory and practice. Int J Teach Learn Higher Educ.

[9] Najafi Kalyani M, Jamshidi N, Molazem Z (2019). How do nursing students experience the clinical learning environment and respond to their experiences? A qualitative study. BMJ Open.

[10] Joolaee S, Jafarian Amiri SR, Farahani MA (2015). Iranian nursing students' preparedness for clinical training: A qualitative study. Nurse Educ Today.

[11] Alluhidan M, Tashkandi N, Alblowi F (2020). Challenges and policy opportunities in nursing in Saudi Arabia. Hum Resour Health.

[12] Aboshaiqah AE, Roco IM, Pandaan IN (2018). Challenges in the Clinical Environment: The Saudi Student Nurses' Experience. Educ Res Int.

[13] Alghamdi R, Albloushi M, Alzahrani E (2019). Nursing Education Challenges from Saudi Nurse Educators' and Leaders' Perspectives: A Qualitative Descriptive Study. Int J Nurs Educ Scholarsh.

[14] Aal M (2015). Clinical nursing teaching in Saudi Arabia challenges and suggested solutions. J Nurs Care S.

[15] Attia YK, Ibrahim RH (2023). Difficulties experienced in clinical learning settings for nurses in Iraq: Perspectives of nursing administrators and nursing instructors. Inform Med Unlocked.

[16] Bivall A-C, Gustavsson M, Lindh Falk A (2021). Conditions for collaboration between higher education and healthcare providers organising clinical placements. High Educ Skill Work.

[17] DeBoer S, Dockx J, Lam C (2019). Building successful and sustainable academic health science partnerships: exploring perspectives of hospital leaders. Can Med Educ J.

[18] Ülker T, Korkmaz F (2017). Collaboration Between Health Care Institutions And Nursing Schools On Clinical Education. Annu World Nurs Conf.

[19] Nachinab GT, Armstrong SJ (2022). Unveiling how Clinical Nursing Education can be Improved in Northern Ghana: The Perspectives of Key Informants. SAGE Open Nurs.

[20] Alsadaan N, Jones LK, Kimpton A (2021). Challenges Facing the Nursing Profession in Saudi Arabia: An Integrative Review. Nurs Rep.

[21] Mussa YM, Muhammed QH, Ali RI (2016). Difficulties of nursing students during clinical training. J Natu Sci Res.

[22] Panda S, Dash M, John J (2021). Challenges faced by student nurses and midwives in clinical learning environment - A systematic review and meta-synthesis. Nurse Educ Today.

[23] Aliafsari Mamaghani E, Rahmani A, Hassankhani H (2018). Experiences of Iranian Nursing Students Regarding Their Clinical Learning Environment. Asian Nurs Res (Korean Soc Nurs Sci).

[24] Ziba FA, Yakong VN, Ali Z (2021). Clinical learning environment of nursing and midwifery students in Ghana. BMC Nurs.

[25] Wong JE, Parikh P, Poh BK (2016). Physical Activity of Malaysian Primary School Children: Comparison by Sociodemographic Variables and Activity Domains. Asia Pac J Public Health.

[26] Mhaidat F (2016). The adaptive problems of female teenage refugees and their behavioral adjustment methods for coping. Psychol Res Behav Manag.

[27] AlHaqwi AI, Taha WS (2015). Promoting excellence in teaching and learning in clinical education. J Taibah Univ Med Sc.

[28] Aljohani KAS (2020). Nursing Education in Saudi Arabia: History and Development. Cureus.

[29] Jamshidi N, Molazem Z, Sharif F (2016). The challenges of nursing students in the clinical learning environment: A qualitative study. Scientific World Iournal.

[30] Bennett LL (2017). The Gap Between Nursing Education and Clinical Skills. ABNF J.

[31] Drateru KC (2019). Challenges Experienced by Student Nurses During Skill Acquisition at The Clinical Area.

[32] Saifan A, Devadas B, Daradkeh F (2021). Solutions to bridge the theory-practice gap in nursing education in the UAE: A qualitative study. BMC Med Edu.

[33] Fawaz MA, Hamdan-Mansour AM, Tassi A (2018). Challenges facing nursing education in the advanced healthcare environment. Int J Africa Nurs Sci.

[34] Younas A, Zeb H, Aziz SB (2019). Perceived challenges of nurse educators while teaching undergraduate nursing students in Pakistan: An exploratory mixed-methods study. Nurse Educ Today.

[35] Jafarian-Amiri SR, Zabihi A, Qalehsari MQ (2020). The challenges of supporting nursing students in clinical education. J Educ Health Promot.

[36] Wright MC, Bergom I, Bartholomew T (2017). Decreased class size, increased active learning? Intended and enacted teaching strategies in smaller classes. Act Learn High Educ.

[37] Hashemiparast M, Negarandeh R, Theofanidis D (2019). Exploring the barriers of utilizing theoretical knowledge in clinical settings: A qualitative study. Int J Nurs Sci.

[38] Busari JO, Moll FM, Duits AJ (2017). Understanding the impact of interprofessional collaboration on the quality of care: a case report from a small-scale resource limited health care environment. J Multidiscip Healthc.

[39] Alanazi MR, Aldhafeeri NA, Salem SS (2023). Clinical environmental stressors and coping behaviors among undergraduate nursing students in Saudi Arabia: A cross-sectional study. Int J Nurs Sci.

[40] Günay U, Kılınç G (2018). The transfer of theoretical knowledge to clinical practice by nursing students and the difficulties they experience: A qualitative study. Nurse Educ Today.

[41] Carless-Kane S, Nowell L (2023). Nursing students learning transfer from classroom to clinical practice: An integrative review. Nurse Educ Pract.

[42] Weldon A, Ma PhD WWK, Ho I (2021). Online learning during a global pandemic: Perceived benefits and issues in higher education. Knowl Manag E-Learn.

[43] Abuosi AA, Kwadan AN, Anaba EA (2022). Number of students in clinical placement and the quality of the clinical learning environment: A cross-sectional study of nursing and midwifery students. Nurse Educ Today.

[44] Stevens KR (2013). The impact of evidence-based practice in nursing and the next big ideas. Online J Issues Nurs.

[45] Baraz S, Memarian R, Vanaki Z (2015). Learning challenges of nursing students in clinical environments: A qualitative study in Iran. J Educ Health Promot.

[46] Hanifi N, Parvizy S, Joolaee S (2012). The Miracle of Communication as a Global Issue in Clinical Learning Motivation of Nursing Students. Procedia Soc Behavi Sci.

[47] Babiker A, El Husseini M, Al Nemri A (2014). Health care professional development: Working as a team to improve patient care. Sudan J Paediatr.

[48] Alamri AA (2022). RN & Sami Alhamidi (2022). Exploring the Barriers and Challenges Saudi Arabian Nursing Students Encounter during their Internship Program: A Qualitative Study. Saudi J Nurs Health Care.

[49] Banafi N (2023). Nursing Students' Knowledge and Attitude Towards Medical Writing Skills in the English Language. J Lang Teach Re.

[50] Magobolo GN, Dube BM (2019). Factors influencing high absenteeism rate of student nurses in clinical areas at a nursing college in the Lejweleputswa District. Curationis.

[51] Akkus M, Cinkir S (2022). The Problem of Student Absenteeism, Its Impact on Educational Environments, and the Evaluation of Current Policies. Int J Psychol Educ Stud.

[52] Sayed SAM, Alsuwat S, Alsufyani J (2022). Factors affecting nursing students learning in clinical from student viewpoint at Taif University. Pakistan J Med Health Sci.

[53] Alotaibi RM, Alkhaldi RM, Turkistani AA (2023). Exploring factors and challenges influencing nursing interns' training experiences in emergency departments in Saudi Arabia. Int Med Educ.

[54] Anarado AN, Agu GU, Nwonu EI (2016). Factors hindering clinical training of students in selected nursing educational institutions in southeastern Nigeria. Nurse Educ Today.

[55] Ergol S, AkyÜZ E (2022). The challenges experienced by nursing students in clinical learning environment and their suggestions. Sağlık ve Hemşirelik Yönetimi Dergisi.

[56] Aljohani W, Banakhar M, Sharif L (2021). Sources of Stress among Saudi Arabian nursing students: A cross-sectional study. Int J Environ Res Public Health.

